# Supplementary material for: The value of angiogenic biomarkers in determining preeclampsia-related perinatal deaths: A cross-sectional study in a high burden setting of southern Mozambique
Source: PLoS One. 2026 Oct 15;21(10):e0339830. doi: 10.1371/journal.pone.0339830 (PMC13671542; doi:10.1371/journal.pone.0339830)
Supplement: Supplementary file 2 [file pone.0339830.s001.docx]

**Supplementary Appendix**

**S1 Table.** **Comparison of maternal, perinatal and placental characteristics of participants with and without PE-associated foetal/neonatal death**

|  | **No preeclampsia related perinatal death**,  N = 37 | **Preeclampsia related perinatal death**,  N = 63 | p-value^4^ |
| --- | --- | --- | --- |
| **Maternal Characteristics** | | | |
| Maternal age | 29.1 (6.6)^1^ | 29.5 (6.8)^1^ | 0.8 |
| Gravidity |  |  | 0.8 |
| *1* | 10 (27.0%)^2^ | 13 (20.6%)^2^ |  |
| *2-4* | 22 (59.5%)^2^ | 40 (63.5%)^2^ |  |
| *≥ 5* | 5 (13.5%)^2^ | 10 (15.9%)^2^ |  |
| GA at delivery (weeks) | 36.4 (31.1, 38.8) ^3^ | 33.3 (30.8, 36.2) ^3^ | 0.016 |
| Mode of delivery |  |  | 0.050 |
| *Vaginal* | 36 (97.3%)^2^ | 53 (84.1%)^2^ |  |
| *Caesarean section* | 1 (2.7%)^2^ | 10 (15.9%)^2^ |  |
| Severe anaemia (Hb<7g/dL) | 0 (0) | 4 (8.0%)^2^ | 0.3 |
| Missing information | 10 | 13 |  |
| Syphilis | 0 (0.0%)^2^ | 1 (1.9%)^2^ | 0.5 |
| Missing information | 1 | 9 |  |
| HIV | 7 (18.9%)^2^ | 9 (14.3%)^2^ | 0.5 |
| Systolic blood pressure, mmHg | 121.0 (118.0, 130.0) ^3^ | 154.0 (139.0, 177.0) ^3^ | <0.001 |
| Diastolic blood pressure, mmHg | 80.0 (75.0, 85.0) ^3^ | 105.0 (96.0, 120.0) ^3^ | <0.001 |
| Preeclampsia | 4 (10.8%)^2^ | 63 (100.0%)^2^ | <0.001 |
| **Perinatal Characteristics** | | | |
| Gestational Age at birth |  |  |  |
| 20-27 weeks | 2 (5.4%)^2^ | 1 (1.6%)^2^ | 0.002 |
| 28-36 weeks | 18 (48.6%)^2^ | 51 (81.0%)^2^ |  |
| ≥ 37 weeks | 17 (45.9%)^2^ | 11 (17.5%)^2^ |  |
| Sex |  |  | 0.4 |
| Female | 11 (29.7%)^2^ | 24 (38.1%)^2^ |  |
| Male | 26 (70.3%)^2^ | 39 (61.9%)^2^ |  |
| Birth Weight (g); median (IQR) | 2525.0 (1420.0, 3195.0) ^3^ | 1450.0 (1057.5, 1935.0) ^3^ | <0.001 |
| Height/length of baby(cm) | 46.0 (41.0, 50.0) ^3^ | 41.0 (38.0, 43.0) ^3^ | <0.001 |
| Head circumference of baby (cm) | 31.0 (29.0, 34.0) ^3^ | 28.0 (25.3, 31.0) ^3^ | 0.007 |
| Preeclampsia as main maternal cause of perinatal death | 0 (0%) ^2^ | 50 (79.4%)^2^ | <0.001 |
| Preeclampsia as main or contributing maternal cause of perinatal death | 0 (0%) | 63 (100.0%)^2^ | <0.001 |
| **Placental characteristics** | | | |
| Placental weight (g) | 310.0 (250.0, 415.0) ^3^ | 195.0 (142.5, 245.0) ^3^ | <0.001 |
| Maximal length of the placental disk | 18.0 (16.0, 19.0) ^3^ | 16.0 (14.0, 17.0) ^3^ | <0.001 |
| Maximal width of the placental disk | 15.0 (14.0, 17.0) ^3^ | 13.0 (12.0, 15.0) ^3^ | <0.001 |
| Length of the umbilical cord | 34.0 (26.5, 44.8) ^3^ | 31.0 (26.0, 37.5) ^3^ | 0.2 |
| Diameter of the umbilical cord (cm) | 2.0 (1.0, 2.0) ^3^ | 1.0 (1.0, 2.0) ^3^ | <0.001 |

^1^ Mean (standard deviation); ^2^ absolute number (percentage); ^3^ median (interquartile range)

^4^ Wilcoxon rank sum test; Pearson’s Chi-squared test; Fisher’s exact test
