# Supplementary material for: The value of angiogenic biomarkers in determining preeclampsia-related perinatal deaths: A cross-sectional study in a high burden setting of southern Mozambique
Source: PLoS One. 2026 Oct 15;21(10):e0339830. doi: 10.1371/journal.pone.0339830 (PMC13671542; doi:10.1371/journal.pone.0339830)
Supplement: Supplementary file 3 [file pone.0339830.s002.docx]

**Supplementary Appendix**

**S2 Table**. **Foetal causes of death identified in the study cases**

|  | **Immediate cause**  **n [% (95 CI)]** | **Comorbid or antecedent cause**  **n (% [95 CI])** |
| --- | --- | --- |
| **Stillbirths (n=98)** |  |  |
| Congenital birth defects^1^ | 2 (2% [0.5 – 7.8]) | 1 (5.2% [0.6 – 32.5]) |
| Congenital Infection | 25 (25% [17.4 – 34.5]) | 8 (42.1% [21.5 – 65.9]) |
| *Pneumonia^2^* | 24 (24% [16.6 – 33.4]) | 4 (21.1% [7.5 – 46.5]) |
| *Cytomegalovirus* | 1 (1% [0.1 – 6.9]) | 4 (21.1% [7.5 – 46.5]) |
| Disorders related to length of gestation and foetal growth^3^ | 1 (1% [0.1 – 6.9]) | 4 (21.1%[7.6 – 46.5]) |
| Umbilical cord complications^4^ | 0 (0) | 2 (10.5% [2.3 – 36.1]) |
| Intrauterine hypoxia^5^ | 70 (70% [60.2 – 78.2]) | 4 (21.1% [7.6 – 46.5]) |
| **Early neonatal deaths (n=2)** |  |  |
| Birth asphyxia | 2 (2% [0.5 – 7.8]) |  |
| **Total** | 100 | 21 |

^1^ One congenital malformation of limbs, one gastroschisis as main fetal cause of death and one congenital CNS malformation as contributing condition to cause of death.

^2^ 22 pneumonia cases had maternal chorioamnionitis and evidence of inflammation in fetal lung tissue and two cases fetal lung inflammation only; the following pathogens were isolated in nine pneumonia cases: *Mycoplasma* (one case), *Streptococcus agalactiae* (seven cases) and *Staphylococcus aureus* (one case).

^3^ fetal macrosomia.

^4^ Cord compression.

^5^ Four babies with meconium aspiration as main cause of death were included in this category
