# Supplementary material for: The value of angiogenic biomarkers in determining preeclampsia-related perinatal deaths: A cross-sectional study in a high burden setting of southern Mozambique
Source: PLoS One. 2026 Oct 15;21(10):e0339830. doi: 10.1371/journal.pone.0339830 (PMC13671542; doi:10.1371/journal.pone.0339830)
Supplement: Supplementary file 4 [file pone.0339830.s003.docx]

**S3 Table.** **Maternal conditions identified according to foetal causes of death**

| **Main perinatal cause of death** | **Main maternal condition** | | | |
| --- | --- | --- | --- | --- |
|  | **M1:**  **Complications of placenta, cord and membranes** | **M4:**  **Maternal medical and surgical conditions** | **M5:**  **No maternal condition** | **Total (%)** |
| Perinatal asphyxia or hypoxia | 13 (37.1) | 52 (98.1) | 7 (53.8) | 72 (72) |
| Congenital infection | 22 (62.9) | 0 | 3 (23.1) | 25 (25) |
| Disorders related to length of gestation & foetal growth | 0 | 0 | 1 (7.7) | 1 (1) |
| Congenital malformations, deformations and chromosomal abnormalities | 0 | 0 | 2 (15.4) | 2 (2) |
| **Total** | 35 | 52 | 13 | 100 |

Values shown are absolute numbers and (percentages)
