# Supplementary material for: The value of angiogenic biomarkers in determining preeclampsia-related perinatal deaths: A cross-sectional study in a high burden setting of southern Mozambique
Source: PLoS One. 2026 Oct 15;21(10):e0339830. doi: 10.1371/journal.pone.0339830 (PMC13671542; doi:10.1371/journal.pone.0339830)
Supplement: Supplementary file 5 [file pone.0339830.s004.docx]

**S4 Table. Proportion of preeclampsia-related stillbirths/early neonatal deaths by sFlt-1/PlGF ratio cut-offs**

| Preeclampsia-related perinatal death^1^ | **sFlt-1/PlGF**^2^ | | | | | |
| --- | --- | --- | --- | --- | --- | --- |
|  | **Maternal blood, n(%)** | | **Placental blood, n(%)** | | **Cord blood, n(%)** | |
|  | **<** 38 | ≥ 38 | **<** 38 | ≥ 38 | **<** 38 | ≥ 38 |
| No | 25 (67.6) | 12 (32.4) | 5 (13.5) | 32 (86.5) | 8 (21.6) | 29 (78.4) |
| Yes | 9 (14.3) | 54 (85.7) | 3 (4.8) | 60 (95.2) | 7 (11.1) | 56 (88.9) |
|  |  |  |  |  |  |  |
|  | **<** 85 | ≥ 85 | **<** 85 | ≥ 85 | **<** 85 | ≥ 85 |
| No | 29 (78.4) | 8 (21.6) | 13 (35.2) | 24 (64.9) | 15 (40.5) | 22 (59.5) |
| Yes | 15 (23.8) | 48 (76.2) | 6 (9.5) | 57 (90.5) | 15 (23.8) | 48 (76.2) |
|  |  |  |  |  |  |  |
|  | **<** 110 | ≥110 | **<** 110 | ≥ 110 | **<** 110 | ≥ 110 |
| No | 30 (81.1) | 7 (18.9) | 18 (48.7) | 19 (51.35) | 19 (51.4)) | 18 (48.7) |
| Yes | 22 (34.9) | 41 (65.1) | 9 (14.3) | 54 (85.7) | 23 (36.5) | 40 (63.5) |
|  |  |  |  |  |  |  |
|  | **Optimal sFlt-1/PlGF**^2^ | | | | | |
|  | **<** 50 | ≥ 50 | **<** 240 | ≥ 240 | **<** 140 | ≥ 140 |
| No | 27 (73) | 10 (15.9) | 29 (78.4) | 8 (21.6) | 24 (64.86) | 13 (35.1) |
| Yes | 10 (15.9) | 53 (84.1) | 21 (33.3) | 42 (66.7) | 26 (41.3) | 37 (58.7) |

^1^ PE-related perinatal death was defined as a death in which PE or Eclampsia were identified as the main or contributing maternal condition leading to the death of the foetus or new-born.

^2^ sFlt-1 - soluble fms-like tyrosine kinase-1, PlGF - Placental Growth Factor
