# Supplementary material for: The value of angiogenic biomarkers in determining preeclampsia-related perinatal deaths: A cross-sectional study in a high burden setting of southern Mozambique
Source: PLoS One. 2026 Oct 15;21(10):e0339830. doi: 10.1371/journal.pone.0339830 (PMC13671542; doi:10.1371/journal.pone.0339830)
Supplement: Supplementary file 6 [file pone.0339830.s005.docx]

**S5 Table. Sensitivity, specificity, PPV, NPV values of optimal sFlt-1/PlGF ratio cutoffs to identify preeclampsia as main cause of perinatal death in study group.**

| **Blood sample** | **Optimal sFlt-1/PIGF cutoff** | **Sensitivity** | **Specificity** | **Accuracy** | **PPV** | **NPV** |
| --- | --- | --- | --- | --- | --- | --- |
|  | | **(% [95% CI])** | | | | |
| Maternal | ≥ 100 | 80 (66 - 90) | 72 (58 - 84) | 76 (66 - 84) | 74 (60 - 85) | 78 (64 - 89) |
| Placental | ≥ 240 | 70 (55 - 82) | 70 (55 - 82) | 70 (60 - 79) | 70 (55 - 82) | 70 (55 - 82) |
| Foetal/neonatal | ≥ 140 | 66 (51 - 79) | 66 (51 - 79) | 66 (56 - 75) | 66 (51 - 79) | 66 (51 - 79) |

Values shown are percentage and 95% confidence intervals

sFlt-1 - soluble fms-like tyrosine kinase-1, PlGF - Placental Growth Factor
