# Supplementary material for: The value of angiogenic biomarkers in determining preeclampsia-related perinatal deaths: A cross-sectional study in a high burden setting of southern Mozambique
Source: PLoS One. 2026 Oct 15;21(10):e0339830. doi: 10.1371/journal.pone.0339830 (PMC13671542; doi:10.1371/journal.pone.0339830)
Supplement: Supplementary file 7 [file pone.0339830.s006.docx]

| **Sample** | **Apparent AUC** | **Bootstrap (In-sample median, 95% CI)** | **Out-of-bag validated AUC (95% CI)** |
| --- | --- | --- | --- |
| **Maternal blood** | 0.82 | 0.82 (0.74 to 0.89) | 0.83 (0.71 to 0.92) |
| **Placental blood** | 0.75 | 0.76 (0.66 to 0.84) | 0.75 (0.63 to 0.87) |

**Supplementary Table S6.** Bootstrap internal validation of the diagnostic performance of the maternal and placental sFlt-1/PlGF ratio models.

AUC: área under the curve; CI: confidence Interval. Bootstrap validation was performed for the maternal and placental blood models, which demonstrated clinically relevant diagnostic performance. The foetal/neonatal model was not further validated because of its limited discriminatory ability (apparent AUC = 0.61).
