# Supplementary material for: The value of angiogenic biomarkers in determining preeclampsia-related perinatal deaths: A cross-sectional study in a high burden setting of southern Mozambique
Source: PLoS One. 2026 Oct 15;21(10):e0339830. doi: 10.1371/journal.pone.0339830 (PMC13671542; doi:10.1371/journal.pone.0339830)
Supplement: Supplementary file 8 [file pone.0339830.s007.docx]

**Supplementary Table S7. Summary of diagnostic checks for multivariable logistic regression models**

| **Model** | **Sample size** | **Events** | **EPV*** | **Maximum VIF** | **Hosmer-Lemeshow *P*** | **Linearity (Box-Tidwell)** | **Model assumptions** |
| --- | --- | --- | --- | --- | --- | --- | --- |
| Maternal blood (sFlt-1/PlGF ≥85) | 99 | 63 | 10.5 | 4.8 | 0.361 | No violation detected | Satisfied |
| Placental blood (sFlt-1/PlGF ≥110) | 99 | 63 | 10.5 | 4.8 | 0.701 | No violation detected | Satisfied |
| Foetal/neonatal blood (sFlt-1/PlGF ≥85) | 99 | 63 | 10.5 | 4.8 | 0.131 | No violation detected | Satisfied |
| Maternal blood (optimal cut-off ≥50) | 99 | 63 | 10.5 | 5.1 | 0.101 | No violation detected | Satisfied |
| Placental blood (optimal cut-off ≥230) | 99 | 63 | 10.5 | 5.0 | 0.634 | No violation detected | Satisfied |
| Foetal/neonatal blood (optimal cut-off ≥140) | 99 | 63 | 10.5 | 4.6 | 0.206 | No violation detected | Satisfied |

EPV, events per variable. Each multivariable model included six predictors, yielding an EPV of 10.5 (63 events/6 predictors), consistent with recommended thresholds for logistic regression model stability. Variance Inflation Factors (VIFs) ranged from 1.1 to 5.1 across models, indicating no evidence of problematic multicollinearity.Linearity of continuous predictors was assessed using the Box-Tidwell test and visual inspection, with no evidence of violation. Model calibration was assessed using the Hosmer-Lemeshow goodness-of-fit test; all models showed adequate fit (P >0.05). Complete-case analysis was performed for all multivariable models.
