# Supplementary material for: The value of angiogenic biomarkers in determining preeclampsia-related perinatal deaths: A cross-sectional study in a high burden setting of southern Mozambique
Source: PLoS One. 2026 Oct 15;21(10):e0339830. doi: 10.1371/journal.pone.0339830 (PMC13671542; doi:10.1371/journal.pone.0339830)
Supplement: Supplementary file 9 [file pone.0339830.s008.docx]

**S1** **Fig. sFlt-1/PlGF ratio concentrations in study participants and women with live births**

^1^ Median (IQR)
