# Supplementary material for: The value of angiogenic biomarkers in determining preeclampsia-related perinatal deaths: A cross-sectional study in a high burden setting of southern Mozambique
Source: PLoS One. 2026 Oct 15;21(10):e0339830. doi: 10.1371/journal.pone.0339830 (PMC13671542; doi:10.1371/journal.pone.0339830)
Supplement: Supplementary file 10 [file pone.0339830.s009.docx]

**S2 Fig. Performance of sFlt-1/PIGF ratio in identifying as main maternal cause of perinatal death in maternal, placental and fetal/neonatal blood.**

AUC- Area under Curve,

sFlt-1 - soluble fms-like tyrosine kinase-1, PlGF - Placental Growth Factor

PE-related perinatal death was defined as a death in which PE or Eclampsia were identified as the main or contributing maternal condition leading to the death of the fetus or newborn.
