# Supplementary material for: The value of angiogenic biomarkers in determining preeclampsia-related perinatal deaths: A cross-sectional study in a high burden setting of southern Mozambique
Source: PLoS One. 2026 Oct 15;21(10):e0339830. doi: 10.1371/journal.pone.0339830 (PMC13671542; doi:10.1371/journal.pone.0339830)
Supplement: Supplementary file 11 [file pone.0339830.s010.docx]

**Placental blood: sFlt-1/PlGF ratio ≥ 110**

|  | OR | 95% CI | p-value | VIF |
| --- | --- | --- | --- | --- |
| sFlt-1/PlGF ratio ≥ 110, placental sample | 6.290 | 2.019, 21.479 | 0.002 | 1.1 |
| Gravidity |  |  |  | 1.2 |
| Primigravidae | — | — |  |  |
| Multigravidae | 2.209 | 0.627, 7.845 | 0.2 |  |
| Birth weight (grams) | 1.001 | 0.999, 1.002 | 0.4 | 4.8 |
| Placental weight (g) | 0.985 | 0.975, 0.994 | 0.002 | 3.3 |
| Diameter of the umbilical cord (cm) | 0.523 | 0.143, 1.857 | 0.3 | 1.4 |
| Abbreviations: CI = Confidence Interval, OR = Odds Ratio, VIF = Variance Inflation Factor | | | | |
| Null deviance = 130; Null df = 98; Log-likelihood = -43.5; AIC = 101; BIC = 119; Deviance = 87.0; Residual df = 92; No. Obs. = 99  Hosmer-Lemeshow Goodness-of-Fit Test: p = 0.701 | | | | |

**Maternal blood: sFlt-1/PlGF ratio ≥ 85**

|  | OR | 95% CI | p-value | VIF |
| --- | --- | --- | --- | --- |
| sFlt-1/PlGF ratio ≥ 85, maternal sample | 8.742 | 2.820, 30.811 | <0.001 | 1.2 |
| Gravidity |  |  |  | 1.1 |
| Primigravidae | — | — |  |  |
| Multigravidae | 3.560 | 0.992, 14.193 | 0.058 |  |
| Birth weight (grams) | 1.001 | 0.999, 1.002 | 0.2 | 4.8 |
| Placental weight (g) | 0.986 | 0.976, 0.995 | 0.003 | 2.9 |
| Diameter of the umbilical cord (cm) | 0.497 | 0.130, 1.810 | 0.3 | 1.4 |
| Abbreviations: CI = Confidence Interval, OR = Odds Ratio, VIF = Variance Inflation Factor | | | | |
| Null deviance = 130; Null df = 98; Log-likelihood = -41.2; AIC = 96.5; BIC = 115; Deviance = 82.5; Residual df = 92; No. Obs. = 99  Hosmer-Lemeshow Goodness-of-Fit Test: p = 0.361 | | | | |

**Fetal/neonatal blood: sFlt-1/PlGF ratio ≥ 85**

|  | OR | 95% CI | p-value | VIF |
| --- | --- | --- | --- | --- |
| sFlt-1/PlGF ratio ≥ 85, fetal/neonatal sample | 1.551 | 0.503, 4.733 | 0.4 | 1.1 |
| Gravidity |  |  |  | 1.1 |
| Primigravidae | — | — |  |  |
| Multigravidae | 3.033 | 0.956, 10.132 | 0.063 |  |
| Birth weight (grams) | 1.000 | 0.999, 1.002 | 0.7 | 4.8 |
| Placental weight (g) | 0.988 | 0.979, 0.996 | 0.005 | 2.8 |
| Diameter of the umbilical cord (cm) | 0.433 | 0.127, 1.413 | 0.2 | 1.4 |
| Abbreviations: CI = Confidence Interval, OR = Odds Ratio, VIF = Variance Inflation Factor | | | | |
| Null deviance = 130; Null df = 98; Log-likelihood = -48.3; AIC = 111; BIC = 129; Deviance = 96.6; Residual df = 92; No. Obs. = 99  Hosmer-Lemeshow Goodness-of-Fit Test: p = 0.131 | | | | |

**Placental blood**

|  | OR | 95% CI | p-value | VIF |
| --- | --- | --- | --- | --- |
| sFlt-1/PlGF ratio ≥ 230, placental sample | 5.982 | 2.029, 19.924 | 0.002 | 1.1 |
| Gravidity |  |  |  | 1.1 |
| Primigravidae | — | — |  |  |
| Multigravidae | 2.877 | 0.855, 10.246 | 0.092 |  |
| Birth weight (grams) | 1.001 | 0.999, 1.002 | 0.2 | 5.0 |
| Placental weight (g) | 0.986 | 0.976, 0.994 | 0.002 | 3.0 |
| Diameter of the umbilical cord (cm) | 0.413 | 0.109, 1.456 | 0.2 | 1.4 |
| Abbreviations: CI = Confidence Interval, OR = Odds Ratio, VIF = Variance Inflation Factor | | | | |
| Null deviance = 130; Null df = 98; Log-likelihood = -43.2; AIC = 100; BIC = 119; Deviance = 86.4; Residual df = 92; No. Obs. = 99  Hosmer-Lemeshow Goodness-of-Fit Test: p = 0.634 | | | | |

**Maternal blood**

|  | OR | 95% CI | p-value | VIF |
| --- | --- | --- | --- | --- |
| sFlt-1/PlGF ratio ≥ 50, maternal sample | 10.580 | 3.370, 37.619 | <0.001 | 1.2 |
| Gravidity |  |  |  | 1.1 |
| Primigravidae | — | — |  |  |
| Multigravidae | 2.781 | 0.754, 11.004 | 0.13 |  |
| Birth weight (grams) | 1.001 | 0.999, 1.002 | 0.2 | 5.1 |
| Placental weight (g) | 0.985 | 0.975, 0.994 | 0.002 | 2.9 |
| Diameter of the umbilical cord (cm) | 0.481 | 0.121, 1.813 | 0.3 | 1.4 |
| Abbreviations: CI = Confidence Interval, OR = Odds Ratio, VIF = Variance Inflation Factor | | | | |
| Null deviance = 130; Null df = 98; Log-likelihood = -40.1; AIC = 94.1; BIC = 112; Deviance = 80.1; Residual df = 92; No. Obs. = 99  Hosmer-Lemeshow Goodness-of-Fit Test: p = 0.101 | | | | |

**Fetal/neonatal blood**

|  | OR | 95% CI | p-value | VIF |
| --- | --- | --- | --- | --- |
| sFlt-1/PlGF ratio ≥ 140, fetal/neonatal sample | 1.228 | 0.428, 3.458 | 0.7 | 1.1 |
| Gravidity |  |  |  | 1.1 |
| Primigravidae | — | — |  |  |
| Multigravidae | 2.895 | 0.925, 9.435 | 0.070 |  |
| Birth weight (grams) | 1.000 | 0.999, 1.002 | 0.5 | 4.6 |
| Placental weight (g) | 0.988 | 0.979, 0.995 | 0.004 | 2.8 |
| Diameter of the umbilical cord (cm) | 0.403 | 0.120, 1.292 | 0.13 | 1.4 |
| Abbreviations: CI = Confidence Interval, OR = Odds Ratio, VIF = Variance Inflation Factor | | | | |
| Null deviance = 130; Null df = 98; Log-likelihood = -48.5; AIC = 111; BIC = 129; Deviance = 97.1; Residual df = 92; No. Obs. = 9  Hosmer-Lemeshow Goodness-of-Fit Test: p = 0.206 | | | | |
